# Vaccine Targets and Antimicrobial Resistance of *Streptococcus agalactiae* Recovered From Pregnant Women in Brazil

**DOI:** 10.1111/tmi.70212

**Published:** 2026-07-30

**Authors:** Clarissa Campos Barros, Fabíola C. Oliveira Kegele, Maria Francisca da S. N. Soares, Douglas Guedes Ferreira, Geraldo Renato de Paula, Rosana Rocha Barros

**Affiliations:** ^1^ Instituto Biomédico, Universidade Federal Fluminense Niterói Rio de Janeiro Brazil; ^2^ Instituto Fernandes Figueira, Fundação Oswaldo Cruz Rio de Janeiro Rio de Janeiro Brazil; ^3^ Hospital Universitário Antônio Pedro, Universidade Federal Fluminense Niterói Rio de Janeiro Brazil; ^4^ Faculdade de Farmácia Universidade Federal Fluminense Niterói Rio de Janeiro Brazil

**Keywords:** bacterial drug resistance, pregnant women, *Streptococcus agalactiae*, vaccines

## Abstract

**Objectives:**

The available measure to prevent *Streptococcus agalactiae* (Group B *Streptococcus,* GBS) neonatal infection is intrapartum antibiotic prophylaxis (IAP), but it is threatened by bacterial resistance. Therefore, this study aimed to investigate antimicrobial resistance and bacterial targets of vaccines at the most advanced stage of development.

**Methods:**

GBS isolates (*n* = 222) recovered from pregnant women between 2019 and 2024 were subjected to capsular polysaccharides and alpha‐like proteins investigation and antimicrobial susceptibility testing. Macrolide and fluoroquinolone resistance determinants and genetic diversity of multidrug‐resistant (MDR) isolates were also investigated.

**Results:**

The main capsular types were Ia (78, 35.1%), III (42, 18.9%), V (39, 17.6%) and II (32, 14.4%). Vaccines based on hexavalent capsular polysaccharides and alpha‐like proteins would have 97.3% and 87.5% of predicted efficacy, respectively. Isolates were categorized as not susceptible (NS), that is, intermediate and resistant, to levofloxacin (5, 2.3%), clindamycin (11, 5%), erythromycin (58, 26.1%) and tetracycline (171, 77%). Among erythromycin NS isolates, the M phenotype/*mef*(A*)* gene predominated (47, 81%). All levofloxacin‐resistant isolates showed amino acid substitutions in both GyrA (S81L) and ParC (S79F). MDR isolates (10, 4.5%) had distinct genetic backgrounds.

**Conclusions:**

Both immunization approaches would be effective in the population. Although the elevated erythromycin resistance rate, one of the highest reported in the country, clindamycin remains an efficient option in IAP. MDR isolates have not resulted from the dissemination of a single clone, but instead showed a polyclonal origin.

## 1 Introduction

*Streptococcus agalactiae* (Group B Streptococcus, GBS) is a pathobiont, a member of the gastrointestinal and genitourinary microbiota that causes diseases in the host. This species is the leading cause of severe neonatal infections, named early‐onset disease (EOD), occurring in the first week of life and late‐onset disease (LOD), between 7 days and 3 months [1]. Maternal colonization is the primary risk factor, and it is globally estimated that up to 36% of pregnant women are colonized by this species [2].

GBS has a diversity of virulence factors, with the polysaccharide capsule, essential for evading the host immune response, being the most relevant. Due to its antigenic variability and association with disease severity, capsule typing represents a valuable epidemiological approach. From the 10 known types, Ia, Ib, II, III and V are found in 98% of isolates recovered from maternal carriage and infection in neonates and adults [1]. An immunization approach based on six capsular polysaccharides (Ia, Ib, II, III, IV and V) has undergone a clinical trial Phase 2 [3].

Other virulence factors, such as the alpha‐like proteins (Alps), are also targets of a vaccine in development [3]. They are surface proteins that mediate adhesion and invasion, found in isolates with different capsular types [4]. Both capsular polysaccharide and Alp vaccines induce the production and transplacental transfer of maternal antibodies to the fetus, preventing fetal death and preterm birth [1, 3].

In the absence of effective maternal vaccines, prevention strategies for GBS neonatal infections rely on intrapartum antibiotic prophylaxis (IAP) of women colonized by GBS at 36 to 37 weeks of gestation [5]. This approach has substantially reduced the incidence of EOD from 1.8 in 1990 to 0.23 infants per 1000 live births in 2015 in the United States [6].

Penicillin is the recommended antibiotic for IAP. However, penicillin reduced susceptibility has been reported since 2008 [7]. Clindamycin and vancomycin are options for penicillin‐allergic patients with risk of anaphylaxis [5]. GBS resistance to clindamycin is a common feature, while resistance to vancomycin remains rare. Despite not being recommended for IAP, macrolide resistance monitoring is relevant due to clindamycin cross‐resistance (MLS phenotypes/*erm* genes). On the other hand, macrolide resistance phenotype M, due to active efflux and encoded by the *mef*(A) gene, does not affect clindamycin. GBS also developed resistance to therapeutic options not involved in IAP, such as fluoroquinolones [8].

In Brazil, the largest country of Latin America, streptococcal neonatal infection is neglected. Such an infection does not have a national compulsory notification, leading to a lack of epidemiological data. In addition, unclear recommendations of public health authorities do not support national GBS screening. While a guideline advocates screening in high‐risk pregnancy [9], this procedure is not recommended for low‐risk pregnant women [10].

Considering the relevance of GBS in maternal and child health, the burden of antibiotic resistance, the need for a reliable prevention approach and the gaps in epidemiological data in Brazil, this study aimed to investigate vaccine targets and antimicrobial resistance of GBS isolates circulating in the country.

## 2 Materials and Methods

### 2.1 Bacterial Isolates

GBS isolates (*n* = 222) were recovered from vaginal secretion (139) and urine (83) of pregnant women during antenatal care in the Instituto Fernandes Figueira and Hospital Universitário Antônio Pedro, from January 2019 to December 2024. Both institutions are reference centers of maternal and neonatal care in the Metropolitan Area of Rio de Janeiro, composed of 22 municipalities and 12 million inhabitants. This study was approved by the local research ethics committee under numbers CAAE 17369913.0.0000.5243 and CAAE 17564819.5.3001.5269.

### 2.2 Investigation of Vaccine Targets

All isolates were submitted to investigation of capsular types (Ia to VIII), and a subset of 112 isolates, recovered in the last years of the study, was submitted to investigation of Rib (*rib*) and Alpha C protein (*bca*) genetic determinants by PCR‐based protocols [11, 12, 13, 14, 15]. DNA was obtained as previously described, with slight modifications [11]. Primers and PCR conditions are those described elsewhere (Table S1). The predictive efficacy of the hexavalent vaccine (Ia, Ib, II, III, IV and V) was estimated by summing the frequencies of capsular types included in its formulation. In the same manner, the predictive efficacy of the alpha‐like protein vaccines (GBS‐NN) was estimated by summing the frequencies of *bca* and *rib* genes.

### 2.3 Antimicrobial Susceptibility Testing (AST)

Isolates were submitted to AST using clindamycin (2 μg), erythromycin (15 μg), levofloxacin (5 μg), penicillin (10 U), tetracycline (30 μg) and vancomycin (30 μg) (CECON, São Paulo, Brazil) by disk diffusion method, according to CLSI guidelines [16]. Macrolide resistance phenotypes were determined by D‐test [16]. Penicillin MIC was determined by epsilometer method (E‐test, bioMérieux, Marcy‐l'Étoile, Auvergne‐Rhône‐Alpes, France). Isolates were classified as multidrug‐resistant (MDR) when resistance to at least three classes of antibiotics was detected [17].

### 2.4 Investigation of Macrolide and Fluoroquinolone Resistance

Erythromycin‐not‐susceptible isolates, that is, intermediate or resistant to this antibiotic, were submitted to investigation of macrolide resistance determinants *erm*(A), *erm*(B) and *mef*(A) by PCR using previous protocols [18, 19]. Primers and PCR conditions are described in Table S1. Levofloxacin‐resistant isolates were investigated by sequencing the internal regions of *gyrA* and *parC* genes [13]. Sequences were edited (Bioedit, http://www.mbio.ncsu.edu/bioedit/bioedit.html) and analyzed (CLC Sequencer Viewer 8.0, Qiagen, Hilden, North Rhine‐Westphalia, Germany), comparing with *gyrA* (https://www.ncbi.nlm.nih.gov/nuccore/LC640632.1) and *parC* (https://www.ncbi.nlm.nih.gov/nuccore/LC649435.1) sequences of reference strain ATCC13813.

### 2.5 Analysis of DNA Restriction Patterns by Pulsed‐Field Gel Electrophoresis (PFGE)

The genetic diversity of MDR isolates was assessed by PFGE. Sample preparation was performed according to previous protocols [20]. DNA band profiles, obtained after *SmaI* digestion (Thermo Fisher Scientific, Waltham, MA, USA), were analyzed by the Gel‐J software [21]. A dendrogram of similarity, based on the Dice coefficient and UPGMA, was generated. A cut‐off of 80% was used to define clonal relationships [22].

### 2.6 Statistical Analysis

Fisher's exact test (https://www.graphpad.com/quickcalcs/contingency1.cfm) was used to assess statistical significance. Given the large, variable sample size and non‐normal distribution of the data, the Bonferroni correction was applied when multiple comparisons were performed (*p* value < 0.05).

## 3 Results

The distribution of capsular types and Rib and Alpha C protein genetic determinants according to clinical source is shown in Table 1. The most frequent capsular types were Ia, III, V and II, which together accounted for 86% of the isolates. Non‐typeable (*n* = 4) isolates were those in which no PCR product was obtained using all nine pairs of primers. Considering the frequency of each type, the hexavalent vaccine's predictive efficacy on this population of pregnant women is 97.3%.

**TABLE 1:** Distribution of capsular types and alpha‐like protein genetic determinants of the isolates according to the specimen.

Specimen	Capsular types (%)								Alpha‐like protein genes (%)			
	Ia	Ib	II	III	IV	V	VI	NT^a^	*bca*	*rib*	*bca/rib*	Neg^b^
Vaginal secretion	45 (32.4)	9 (6.5)	23 (16.5)	22 (15.8)	8 (5.8)	27 (19.4)	2 (1.4)	3 (2.2)	29 (36.3)	39 (48.8)	4 (5)	8 (10)
Urine	33 (39.8)	4 (4.8)	9 (10.8)	20 (24.1)	4 (4.8)	12 (14.5)	0	1 (1.2)	10 (31.3)	16 (50)	0	6 (18.8)
Total	78 (35.1)	13 (5.9)	32 (14.4)	42 (18.9)	12 (5.4)	39 (17.6)	2 (0.9)	4 (1.8)	39 (34.8)	55 (49.1)	4 (3.6)	14 (12.5)

^a^ NT, non‐typeable.

^b^ Neg, negative.

The distribution of *bca*, *rib* or both genes among isolates with different capsular types is shown in Table 2. Considering the frequencies of such genes, *bca* (34.8%), *rib* (49.1%) and both (3.6%), the GBS‐NN vaccine would have a predictive efficacy of 87.5%. The *rib* gene was associated with types Ia (32/38; 84.2%) and III (12/13; 92.3%), while *bca* was associated with types Ib (9/11; 81.8%), IV (11/11; 100%) and V (15/20; 75%) (*p* < 0.00625).

**TABLE 2:** Frequency of *bca, rib* or both genes in GBS isolates of different capsular types.

Gene	Capsular type (%)							
	Ia	Ib	II	III	IV	V	VI	NT
*bca*	0	72.7	20	7.7	100	65	100	50
*rib*	81.6	0	66.7	92.3	0	10	0	0
*bca/rib*	2.6	9.1	0	0	0	10	0	0
Negative	15.8	18.2	13.3	0	0	15	0	50

Abbreviation: NT, non‐typeable.

All isolates were susceptible to vancomycin and penicillin, the latter with MIC values ranging from 0.012 to 0.064 μg/mL. Rates of antimicrobial not susceptible, that is, intermediate and resistant isolates, and macrolide resistance phenotypes and genotypes are shown in Table 3 and Figure 1. All levofloxacin‐resistant isolates showed point mutations in *gyrA* and *parC* genes, leading to S81L in GyrA and S79F in ParC.

**TABLE 3:** Antibiotic‐resistant isolates, macrolide‐resistance phenotypes and genotypes and capsular types of erythromycin not susceptible isolates.

Year	Number of isolates	Tet^a^ NS^b^ isolates (%)	Levo^c^ R^d^ isolates (%)	Erythromycin NS isolates (%)	Clindamycin R isolates (%)	Macrolide resistance genotypes and capsular types according to phenotype		
						M (n)	iMLS_B_ (n)	cMLS_B_ (n)
2019	16	15 (93.8)	0	4 (25)	0	*mef(A)* (4) Ia (3), III (1)	0	0
2020	24	20 (83.3)	0	5 (20.8)	2 (8.3)	*mef(A)* (3) Ia (2), III (1)	0	*erm(B)* (2) Ia (1), II (1)
2021	22	15 (68.2)	1 (4.5)	6 (27.3)	1 (4.5)	*mef(A)* (5) Ia (5)	*erm(A)*, V (1)	0
2022	53	39 (73.6)	2 (3.8)	12 (22.6)	3 (5.7)	*mef(A)* (9) Ia (5), II (2), III (2)	*erm(A)* (1), *erm(A)*/*mef(A)* (1) V (2)	*erm(B)*, II (1)
2023	59	49 (83.1)	1 (1.7)	18 (30.5)	1 (17)	*mef(A)* (16) Ia (11), III (2), V (2), IV (1)	*erm(A)*, Ib (1)	0
2024	48	33 (68.8)	1 (2.1)	13 (27)	4 (8.3)	*mef(A)* (9) Ia (7), Ib (2)	*erm(A)* (2) Ib (1), V (1)	*erm(B)* (2) VI (1), NT^e^ (1)
Total	222	171 (77)	5 (2.3)	58 (26.1)	11 (5)	47	6	5

^a^ Tet, tetracycline.

^b^ NS, not susceptible, that is, intermediate or resistant.

^c^ Levo, levofloxacin.

^d^ R, resistant.

^e^ NT, non‐typeable.

**FIGURE 1:**
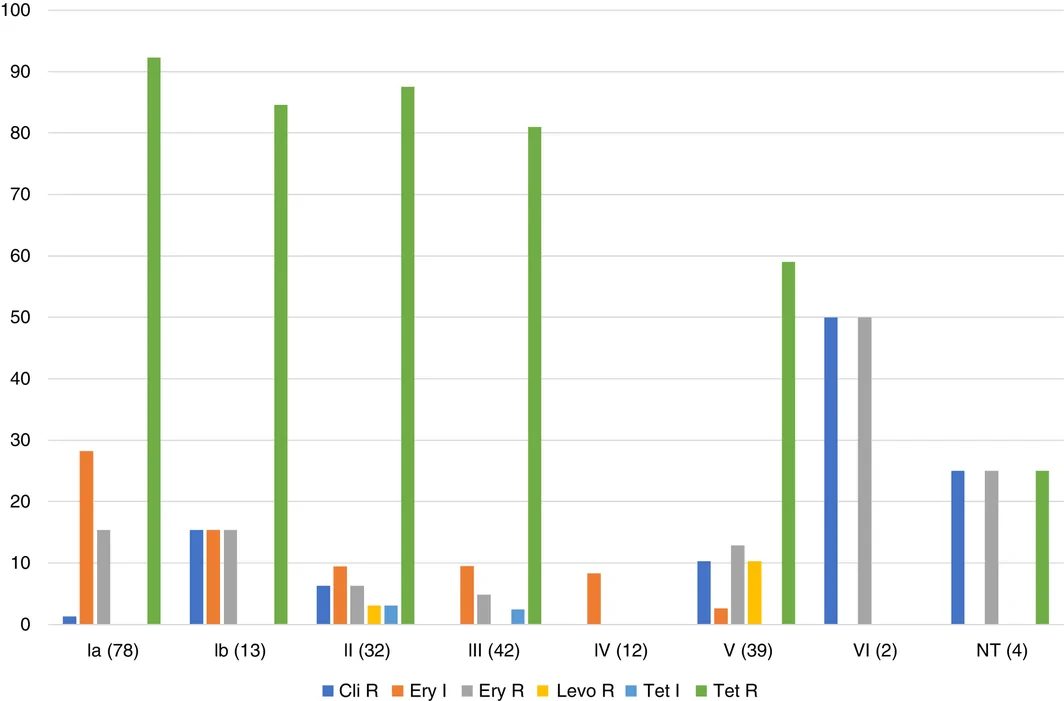
Frequency of intermediate and resistant isolates to the tested antibiotics.

Ten isolates (4.5%) classified as MDR were resistant to at least three of the antibiotics: clindamycin, erythromycin, levofloxacin and tetracycline. The PFGE profiles, dendrogram and characteristics of MDR isolates are shown in Figure 2. Similarity of DNA profiles ranged from 51% to 100%. Three of four capsular type V isolates yielded indistinguishable profiles. Two of three capsular type II isolates were clustered in a clonal group (similarity > 80%), as well as both type Ib isolates (similarity > 90%). No clonal group was observed among isolates with different capsular types.

**FIGURE 2:**
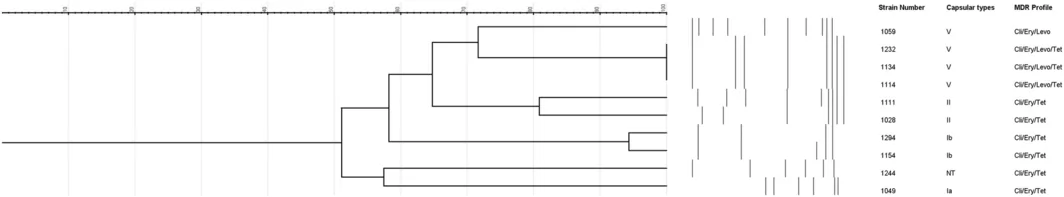
Dendrogram generated from DNA band profiles after *Sma*I digestion and characteristics of MDR isolates.

## 4 Discussion

Despite the lack of national data on streptococcal neonatal infections, several studies, mainly in academia, have investigated the distribution of GBS capsular types in the context of neonatal infection and maternal colonization in Brazil [20, 23, 24]. The country has a peculiar prevalence of type Ia in both colonization and infection, as found here and in other national studies [20, 23, 24]. Two isolates were identified as type VI, detected for the first time in Brazil in 2017 [25]. Capsular polysaccharide type VI is not a component of the hexavalent vaccine under development. Therefore, continuous surveillance is required to monitor the overall distribution of capsular types and detect these uncommon types within the circulating bacterial population, especially from the perspective of a future capsular type‐based vaccine.

Regarding the Alp family‐based vaccine, the current formulation in development is composed not only of Rib and Alpha C (GBS‐NN), but also of Alp1 and Alp2/3 proteins (GBS‐NN2) [3]. A limitation of this study was not having investigated the genetic determinants of GBS‐NN2 components; therefore, the predictive efficacy might be higher than observed. The prevalence of Alp genetic determinants varies according to the bacterial population studied. While the prevalence of *bca* here was 38.4%, a recent study from the North region of Brazil found 84.7% [26]. Studies from other countries also found variable rates of *bca*, from 22% to 66% [27, 28]. These same studies found rates of *rib* ranging from 32% to 40%. Here, we found a significant association between *rib* and capsular types Ia and III. The association of *rib* with type III was previously observed [27, 28, 29]. However, our findings differ from the literature regarding capsular type Ia, traditionally associated with *bca* [29]. The association of capsular type Ib and *bca* found here has also been reported [27, 29].

In this study, all isolates were susceptible to penicillin and vancomycin. Resistance to these agents has not been reported in Brazil [30], but it is documented in other countries [31]. On the other hand, high rates of resistance to tetracycline, as found here, have been reported in several national studies and elsewhere [30, 31]. Although not recommended for GBS human infections, tetracycline is widely used in veterinary medicine, where this species is an important pathogen, underscoring the importance of resistance monitoring [32].

In Brazil, GBS resistance to levofloxacin does not exceed 7% [30]. Therefore, the rate observed here shows no increase over time, while in other regions fluoroquinolone resistance reaches 70% [31]. All levofloxacin‐resistant isolates presented amino acid substitutions previously described [13]. Regarding erythromycin, the rate of not susceptible isolates is among the highest reported in national studies [24, 26], but lower than reported in other regions, whose rates are superior to 60% [31]. Here, the prevalence of isolates exhibiting the M phenotype allows the use of clindamycin in IAP. In fact, the M phenotype/*mef*(A) gene has recently become the commonest resistance determinant among GBS isolates circulating in the country [24, 26].

Despite the absence of penicillin reduced susceptibility, resistance to other IAP options, such as clindamycin, as well as to erythromycin, levofloxacin and/or tetracycline, characterized the MDR isolates. Considering that GBS penicillin and clindamycin resistance is a global concern [33, 34], continuous monitoring of antimicrobial resistance among GBS recovered in diverse settings is essential. MDR isolates were submitted to PFGE analysis, which revealed a broad genetic diversity, demonstrating the spread of resistance determinants in distinct lineages. On the other hand, identical DNA profiles or clonal groups were observed among isolates within the same capsular type. GBS capsular switching, a rare phenomenon [23], was not detected in this small set of MDR isolates.

In conclusion, our findings highlight the importance of incorporating GBS screening with antimicrobial susceptibility testing into prenatal care programs to ensure effective intrapartum prophylaxis. In general, Brazil has lower rates of antibiotic‐resistant GBS compared with other regions. Despite the erythromycin resistance rate being among the highest reported in the country, the major phenotypic mechanism does not impact clindamycin efficacy, allowing its use for IAP. The findings of MDR isolates of distinct lineages demonstrate the widespread occurrence of such genetic determinants. On the other hand, the impact of both immunization approaches, based on capsular polysaccharides and alpha‐like proteins, would be relevant in the local population of pregnant women.

## Funding

This work was supported by the Fundação Carlos Chagas Filho de Amparo à Pesquisa do Estado do Rio de Janeiro under Grant (APQ1 E‐26/211.681/2021) and Coordenação de Aperfeiçoamento de Pessoal de Nível Superior—Brazil (CAPES) under Grant 001.

## Conflicts of Interest

The authors declare no conflicts of interest.

## Supporting information


**Table S1:** Oligonucleotide primer pairs used in the study.

## Data Availability

Data sharing not applicable to this article as no datasets were generated or analysed during the current study.
