# Supplementary material for: Vaccine Targets and Antimicrobial Resistance of Streptococcus agalactiae Recovered From Pregnant Women in Brazil
Source: Trop Med Int Health. 2026 Jul 30;31(11):1701–7. doi: 10.1111/tmi.70212 (PMC13727695; doi:10.1111/tmi.70212)
Supplement: Supplementary file 1 [file TMI-31-1701-s001.docx]

Table S1: oligonucleotide primer pairs used in the study

| Primer name | Gene target | Sequence (5’-3’) | Amplicon size (bp) | Annealing temp. (^o^C) | Reference |
| --- | --- | --- | --- | --- | --- |
| bca F | *bca* | CAGAGTACAGGAAGGGCTAGTC | 183 | 50 | Dmitriev et al., 2002 |
| bca R |  | TTCTTCCGTCCACTTAGGATC |  |  |  |
| Universal F | *rib* | TGATACTTCACAGACGAAACAACG | 295 | 50 | Creti et al., 2004 |
| Rib R |  | CATACTGAGCTTTTAAATCAGGTGA |  |  |  |
| Ia-F | *cps1aH* | GGTCAGACTGGATTAATGGTATGC | 521 and 1826 | 52 | Poyart et al., 2007 |
| Ia-R |  | GTAGAAATAGCCTATATACGTTGAATGC |  |  |  |
| Ib-F | *cps1bJ* | TAAACGAGAATGGAATATCACAAACC | 770 | 52 | Poyart et al., 2007 |
| Ib-R | *cps1bK* | GAATTAACTTCAATCCCTAAACAATATCG |  |  |  |
| II-F | *cps2K* | GCTTCAGTAAGTATTGTAAGACGATAG | 397 | 52 | Poyart et al., 2007 |
| II-R |  | TTCTCTAGGAAATCAAATAATTCTATAGGG |  |  |  |
| III-R | *cps1a/2/3I* | TCCGTACTACAACAGACTCATCC | 1826 | 52 | Poyart et al., 2007 |
| III-F | *cps1a/2/3J* | AGTAACCGTCCATACATTCTATAAGC |  |  |  |
| IV-F | *cps4N* | GGTGGTAATCCTAAGAGTGAACTGT | 578 | 52 | Poyart et al., 2007 |
| IV-R |  | CCTCCCCAATTTCGTCCATAATGGT |  |  |  |
| V-F | *cps5O* | GAGGCCAATCAGTTGCACGTAA | 701 | 52 | Poyart et al., 2007 |
| V-R |  | AACCTTCTCCTTCACACTAATCCT |  |  |  |
| VI-F | *cps6I* | GGACTTGAGATGGCAGAAGGTGAA | 487 | 52 | Poyart et al., 2007 |
| VI-R |  | CTGTCGGACTATCCTGATGAATCTC |  |  |  |
| VII-F | *cps7M* | CCTGGAGAGAACAATGTCCAGAT | 371 | 52 | Poyart et al., 2007 |
| VII-R |  | GCTGGTCGTGATTTCTACACA |  |  |  |
| VIII-F | *cps8J* | AGGTCAACCACTATATAGCGA | 282 | 52 | Poyart et al., 2007 |
| VIII-R |  | TCTTCAAATTCCGCTGACTT |  |  |  |
| dlts-F | *dltS* | CTGTAAGTCTTTATCTTTCTCG | 199 | 52 | Murayama et al., 2009 |
| dlts-R |  | TCCATTCGCTTAGTCTCC |  |  |  |
| mef F | *mef* | AGTATCATTAATCACTAGTGC | 345 | 50 | Perez-Trallero et al., 2007 |
| mef R |  | TTCTTCTGGTACTAAAAGTGG |  |  |  |
| ermA F | *ermA* | AACTTGTGGAAATGAGTCAACGG | 375 | 50 | Perez-Trallero et al., 2007 |
| ermA R |  | CAGAATCTACATTAGGCTTAGGG |  |  |  |
| Primer name | Gene target | Sequence (5’-3’) | Amplicon size (bp) | Annealing temp. (^o^C) | Reference |
| ermB F | *ermB* | GAAAAGGTACTCAACCAAATA | 639 | 50 | Sutcliffe et al., 1996 |
| ermB R |  | CATTTGTTAAATTCATGGCAATGA |  |  |  |
| gyrA-F | *gyrA* | GGTTTAAAACCTGTTCATCGTCGT | 407 | 52 | Murayama et al., 2009 |
| gyrA-R |  | GCAATACCAGTTGCACCATTGACT |  |  |  |
| parC-F | *parC* | CCGGATATTCGTGATGGCTT | 403 | 52 | Murayama et al., 2009 |
| parC-R |  | TGACTAAAAGATTGGGAAAGGC |  |  |  |
